# *N*,*N*′-[4,4′-Methyl­enebis(4,1-phenyl­ene)]bis­(2,6-difluoro­benzamide)

**DOI:** 10.1107/S1600536811024524

**Published:** 2011-06-30

**Authors:** Mohammad T. M. Al-Dajani, Jamal Talaat, Nornisah Mohamed, Madhukar Hemamalini, Hoong-Kun Fun

**Affiliations:** a School of Pharmaceutical Sciences, Universiti Sains Malaysia, 11800 USM, Penang, Malaysia; b Virginia Commonwealth University, Chemistry School, USA, Malaysia; c X-ray Crystallography Unit, School of Physics, Universiti Sains Malaysia, 11800 USM, Penang, Malaysia

## Abstract

The complete mol­ecule of the title compound, C_27_H_18_F_4_N_2_O_2_, is generated by crystallographic twofold symmetry, with one C atom lying on the rotation axis. The dihedral angle between fluoro-substituted phenyl ring and the adjacent benzene ring is 10.37 (5)°. In the crystal, mol­ecules are connected by N—H⋯O and C—H⋯F hydrogen bonds, resulting in supra­molecular chains propagating along the *c* direction.

## Related literature

For applications of benzamide derivatives, see: Ashwood *et al.* (1990 ▶); Kees *et al.* (1989 ▶); Ragavan *et al.* (2010 ▶); Carmellino *et al.* (1994 ▶); Rauko *et al.* (2001 ▶). For a related structure, see: Cronin *et al.* (2000 ▶). For the stability of the temperature controller used in the data collection, see: Cosier & Glazer (1986 ▶).
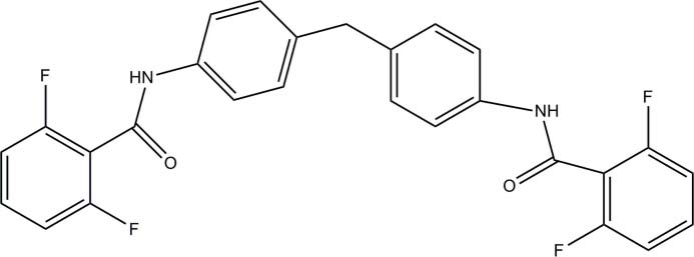


## Experimental

### 

#### Crystal data

C_27_H_18_F_4_N_2_O_2_
*M* *_r_* = 478.43
Monoclinic, 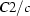

*a* = 42.0478 (10) Å
*b* = 5.2980 (1) Å
*c* = 9.5643 (2) Å
β = 92.522 (2)°
*V* = 2128.57 (8) Å^3^
*Z* = 4
Mo *K*α radiation
μ = 0.12 mm^−1^
*T* = 100 K
0.48 × 0.38 × 0.05 mm

#### Data collection

Bruker APEXII DUO CCD diffractometer
Absorption correction: multi-scan (*SADABS*; Bruker, 2009 ▶) *T*_min_ = 0.946, *T*_max_ = 0.994
26445 measured reflections
3871 independent reflections
3172 reflections with *I* > 2σ(*I*)
*R*_int_ = 0.031

#### Refinement

*R*[*F* ^2^ > 2σ(*F* ^2^)] = 0.040
*wR*(*F* ^2^) = 0.114
*S* = 1.06
3871 reflections
163 parameters
H atoms treated by a mixture of independent and constrained refinement
Δρ_max_ = 0.43 e Å^−3^
Δρ_min_ = −0.21 e Å^−3^

### 

Data collection: *APEX2* (Bruker, 2009 ▶); cell refinement: *SAINT* (Bruker, 2009 ▶); data reduction: *SAINT*; program(s) used to solve structure: *SHELXTL* (Sheldrick, 2008 ▶); program(s) used to refine structure: *SHELXTL*; molecular graphics: *SHELXTL*; software used to prepare material for publication: *SHELXTL* and *PLATON* (Spek, 2009 ▶).

## Supplementary Material

Crystal structure: contains datablock(s) global, I. DOI: 10.1107/S1600536811024524/hb5924sup1.cif

Structure factors: contains datablock(s) I. DOI: 10.1107/S1600536811024524/hb5924Isup2.hkl

Supplementary material file. DOI: 10.1107/S1600536811024524/hb5924Isup3.cml

Additional supplementary materials:  crystallographic information; 3D view; checkCIF report

## Figures and Tables

**Table 1:** Hydrogen-bond geometry (Å, °)

*D*—H⋯*A*	*D*—H	H⋯*A*	*D*⋯*A*	*D*—H⋯*A*
N1—H1*N*1⋯O1^i^	0.841 (15)	2.078 (15)	2.8811 (11)	159.4 (14)
C9—H9*A*⋯F1^ii^	0.95	2.41	3.2318 (11)	145

Symmetry codes: (i) 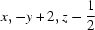; (ii) 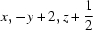.

**Fig. 1.**
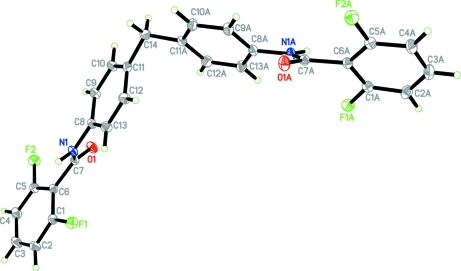
The asymmetric unit of the title compound, showing 50% probability displacement ellipsoids.

**Fig. 2.**
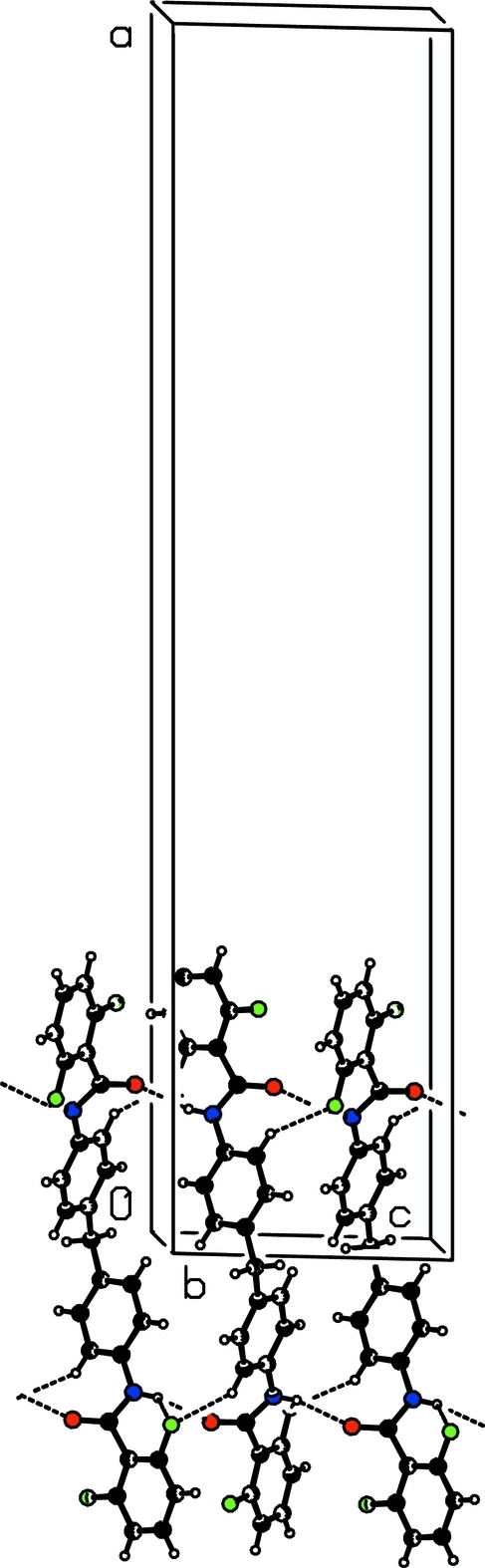
A view of a one-dimensional supramolecular chain along the c-axis

## supplementary crystallographic information

### Comment

A number of benzamide derivatives were reported to possess anti-hypertensive (Ashwood *et al.*, 1990), anti-diabetic (Kees *et al.*, 1989), anti-bacterial (Ragavan *et al.*, 2010), anti-fungal (Carmellino *et al.*, 1994) and anti-cancer (Rauko *et al.*, 2001) activities.  As a part of our study on the synthesis of new fluorine- containing compounds with possible biological activities, we report here the crystal structure of the title compound, (I).

The asymmetric unit of the title compound, (Fig. 1), consists of a half molecule of *N,N'*-(4,4'-methylenebis(4,1-phenylene))bis (2,6-difluorobenzamide), which has a twofold symmetry and it adopts an *E, E* conformation.  The dihedral angle between fluoro- substituted phenyl (C1–C6) rings and benzene (C8–13) ring is 10.37 (5)°. All bond lengths and angles are comparable to values observed in a closely related benzamide structure (Cronin *et al.*, 2000).

In the crystal structure (Fig. 2), the adjacent molecules are connected *via* N1—H1N1..O1 and C9—H9A···F1 (Table 1) hydrogen bonds forming one-dimensional supramolecular chains along the *c*-axis.

### Experimental

In a round bottom flask, 25ml of tetrahydrofuran (THF) was mixed with 4,4'-diaminodiphenylmethane (0.01 mol,2 g) with stirring. Drops of 2,6-Difluorobenzylchloride (0.02 mol, 3.4 g ) which was dissolved in THF was then added. The mixture was refluxed for 30 min and the yellow precipitate formed was filtered and washed with alkaline water, then with toluene and further washed with dilute hydrochloric acid. The precipate was dissolved in methanol at room temperature yielding colourless plates of (I).

### Refinement

Atom H1N1 was located from a difference Fourier maps and refined freely [N–H = 0.842 (15) Å]. The remaining H atoms were positioned geometrically [C–H = 0.95–0.9601 Å] and were refined using a riding model, with *U*_iso_(H) = 1.2 *U*_eq_(C). The highest residual electron density peak is located at 0.61 Å from C6 and the deepest hole 0.64 Å located at from C1.

### Crystal data

**Table:**

C_27_H_18_F_4_N_2_O_2_	*F*(000) = 984
*M**_r_* = 478.43	*D*_x_ = 1.493 Mg m^−^^3^
Monoclinic, *C*2/*c*	Mo *K*α radiation, λ = 0.71073 Å
Hall symbol: -C 2yc	Cell parameters from 8910 reflections
*a* = 42.0478 (10) Å	θ = 2.9–32.5°
*b* = 5.2980 (1) Å	µ = 0.12 mm^−^^1^
*c* = 9.5643 (2) Å	*T* = 100 K
β = 92.522 (2)°	Plate, colourless
*V* = 2128.57 (8) Å^3^	0.48 × 0.38 × 0.05 mm
*Z* = 4	

### Data collection

**Table:**

Bruker APEXII DUO CCD diffractometer	3871 independent reflections
Radiation source: fine-focus sealed tube	3172 reflections with *I* > 2σ(*I*)
graphite	*R*_int_ = 0.031
φ and ω scans	θ_max_ = 32.6°, θ_min_ = 1.9°
Absorption correction: multi-scan (*SADABS*; Bruker, 2009)	*h* = −63→63
*T*_min_ = 0.946, *T*_max_ = 0.994	*k* = −7→7
26445 measured reflections	*l* = −14→14

### Refinement

**Table:**

Refinement on *F*^2^	Primary atom site location: structure-invariant direct methods
Least-squares matrix: full	Secondary atom site location: difference Fourier map
*R*[*F*^2^ > 2σ(*F*^2^)] = 0.040	Hydrogen site location: inferred from neighbouring sites
*wR*(*F*^2^) = 0.114	H atoms treated by a mixture of independent and constrained refinement
*S* = 1.06	*w* = 1/[σ^2^(*F*_o_^2^) + (0.0569*P*)^2^ + 1.3081*P*] where *P* = (*F*_o_^2^ + 2*F*_c_^2^)/3
3871 reflections	(Δ/σ)_max_ < 0.001
163 parameters	Δρ_max_ = 0.43 e Å^−^^3^
0 restraints	Δρ_min_ = −0.21 e Å^−^^3^

### Special details

**Table:**

Experimental. The crystal was placed in the cold stream of an Oxford Cryosystems Cobra open-flow nitrogen cryostat (Cosier & Glazer, 1986) operating at 100.0 (1) K.
Geometry. All s.u.'s (except the s.u. in the dihedral angle between two l.s. planes) are estimated using the full covariance matrix. The cell s.u.'s are taken into account individually in the estimation of s.u.'s in distances, angles and torsion angles; correlations between s.u.'s in cell parameters are only used when they are defined by crystal symmetry. An approximate (isotropic) treatment of cell s.u.'s is used for estimating s.u.'s involving l.s. planes.
Refinement. Refinement of F^2^ against ALL reflections. The weighted R-factor wR and goodness of fit S are based on F^2^, conventional R-factors R are based on F, with F set to zero for negative F^2^. The threshold expression of F^2^ > 2σ(F^2^) is used only for calculating R-factors(gt) etc. and is not relevant to the choice of reflections for refinement. R-factors based on F^2^ are statistically about twice as large as those based on F, and R- factors based on ALL data will be even larger.

### Fractional atomic coordinates and isotropic or equivalent isotropic displacement parameters (Å^2^)

**Table:**

	*x*	*y*	*z*	*U*_iso_*/*U*_eq_	
F1	0.131371 (14)	0.52803 (12)	0.04350 (6)	0.02016 (14)	
F2	0.201443 (15)	1.06377 (13)	0.30050 (7)	0.02303 (15)	
O1	0.136883 (18)	0.97437 (15)	0.36217 (7)	0.02009 (16)	
N1	0.113987 (19)	1.00956 (16)	0.14156 (8)	0.01541 (16)	
C1	0.16118 (2)	0.60034 (18)	0.08307 (9)	0.01554 (17)	
C2	0.18629 (3)	0.4631 (2)	0.03344 (10)	0.01989 (19)	
H2A	0.1826	0.3219	−0.0264	0.024*	
C3	0.21695 (2)	0.5379 (2)	0.07365 (11)	0.0223 (2)	
H3A	0.2346	0.4482	0.0398	0.027*	
C4	0.22225 (2)	0.7422 (2)	0.16280 (11)	0.0207 (2)	
H4A	0.2433	0.7941	0.1895	0.025*	
C5	0.19617 (2)	0.86802 (19)	0.21155 (10)	0.01656 (18)	
C6	0.16482 (2)	0.80494 (17)	0.17353 (9)	0.01396 (16)	
C7	0.13715 (2)	0.93935 (17)	0.23513 (9)	0.01430 (17)	
C8	0.08569 (2)	1.13880 (18)	0.17534 (9)	0.01475 (17)	
C9	0.08638 (2)	1.33660 (19)	0.27115 (10)	0.01791 (18)	
H9A	0.1058	1.3826	0.3189	0.021*	
C10	0.05852 (2)	1.46660 (19)	0.29678 (10)	0.01816 (18)	
H10A	0.0591	1.6004	0.3630	0.022*	
C11	0.02971 (2)	1.40427 (18)	0.22709 (10)	0.01674 (18)	
C12	0.02942 (2)	1.20383 (19)	0.13243 (11)	0.01962 (19)	
H12A	0.0100	1.1570	0.0852	0.024*	
C13	0.05708 (2)	1.07101 (19)	0.10588 (10)	0.01794 (18)	
H13A	0.0565	0.9352	0.0409	0.022*	
C14	0.0000	1.5573 (3)	0.2500	0.0213 (3)	
H14A	−0.0040	1.6645	0.1702	0.026*	
H1N1	0.1173 (3)	0.984 (3)	0.0566 (16)	0.023 (4)*	

### Atomic displacement parameters (Å^2^)

**Table:**

	*U*^11^	*U*^22^	*U*^33^	*U*^12^	*U*^13^	*U*^23^
F1	0.0186 (3)	0.0209 (3)	0.0208 (3)	−0.0022 (2)	−0.0022 (2)	−0.0022 (2)
F2	0.0207 (3)	0.0257 (3)	0.0225 (3)	−0.0035 (2)	−0.0013 (2)	−0.0072 (2)
O1	0.0220 (3)	0.0272 (4)	0.0112 (3)	0.0051 (3)	0.0022 (2)	0.0005 (3)
N1	0.0144 (3)	0.0210 (4)	0.0110 (3)	0.0032 (3)	0.0022 (3)	−0.0003 (3)
C1	0.0159 (4)	0.0180 (4)	0.0127 (4)	0.0003 (3)	0.0000 (3)	0.0020 (3)
C2	0.0249 (5)	0.0197 (5)	0.0151 (4)	0.0056 (4)	0.0019 (3)	−0.0017 (3)
C3	0.0201 (4)	0.0269 (5)	0.0202 (4)	0.0078 (4)	0.0043 (4)	0.0006 (4)
C4	0.0148 (4)	0.0268 (5)	0.0205 (4)	0.0026 (3)	0.0012 (3)	0.0017 (4)
C5	0.0168 (4)	0.0189 (4)	0.0139 (4)	−0.0001 (3)	−0.0001 (3)	−0.0001 (3)
C6	0.0146 (4)	0.0161 (4)	0.0112 (3)	0.0008 (3)	0.0011 (3)	0.0009 (3)
C7	0.0150 (4)	0.0151 (4)	0.0130 (4)	0.0003 (3)	0.0022 (3)	0.0010 (3)
C8	0.0137 (4)	0.0176 (4)	0.0132 (4)	0.0012 (3)	0.0024 (3)	0.0011 (3)
C9	0.0151 (4)	0.0220 (4)	0.0166 (4)	0.0002 (3)	0.0003 (3)	−0.0031 (3)
C10	0.0166 (4)	0.0194 (4)	0.0186 (4)	0.0005 (3)	0.0023 (3)	−0.0036 (3)
C11	0.0139 (4)	0.0158 (4)	0.0208 (4)	0.0000 (3)	0.0042 (3)	0.0014 (3)
C12	0.0145 (4)	0.0198 (4)	0.0244 (5)	−0.0008 (3)	−0.0001 (3)	−0.0023 (4)
C13	0.0166 (4)	0.0185 (4)	0.0187 (4)	0.0000 (3)	0.0004 (3)	−0.0032 (3)
C14	0.0140 (6)	0.0159 (6)	0.0343 (8)	0.000	0.0044 (5)	0.000

### Geometric parameters (Å, °)

**Table:**

F1—C1	1.3489 (11)	C6—C7	1.5058 (13)
F2—C5	1.3534 (11)	C8—C9	1.3916 (13)
O1—C7	1.2297 (11)	C8—C13	1.3954 (13)
N1—C7	1.3459 (12)	C9—C10	1.3901 (13)
N1—C8	1.4222 (12)	C9—H9A	0.9500
N1—H1N1	0.842 (15)	C10—C11	1.3963 (13)
C1—C2	1.3828 (13)	C10—H10A	0.9500
C1—C6	1.3911 (13)	C11—C12	1.3952 (14)
C2—C3	1.3870 (15)	C11—C14	1.5131 (12)
C2—H2A	0.9500	C12—C13	1.3922 (13)
C3—C4	1.3899 (15)	C12—H12A	0.9500
C3—H3A	0.9500	C13—H13A	0.9500
C4—C5	1.3815 (13)	C14—C11^i^	1.5131 (12)
C4—H4A	0.9500	C14—H14A	0.9601
C5—C6	1.3929 (12)		
			
C7—N1—C8	124.77 (8)	N1—C7—C6	114.80 (8)
C7—N1—H1N1	116.8 (10)	C9—C8—C13	119.99 (9)
C8—N1—H1N1	118.2 (10)	C9—C8—N1	121.25 (8)
F1—C1—C2	117.93 (9)	C13—C8—N1	118.70 (8)
F1—C1—C6	118.10 (8)	C10—C9—C8	119.73 (9)
C2—C1—C6	123.96 (9)	C10—C9—H9A	120.1
C1—C2—C3	117.96 (9)	C8—C9—H9A	120.1
C1—C2—H2A	121.0	C9—C10—C11	121.26 (9)
C3—C2—H2A	121.0	C9—C10—H10A	119.4
C2—C3—C4	120.98 (9)	C11—C10—H10A	119.4
C2—C3—H3A	119.5	C12—C11—C10	118.18 (9)
C4—C3—H3A	119.5	C12—C11—C14	121.20 (8)
C5—C4—C3	118.33 (9)	C10—C11—C14	120.57 (8)
C5—C4—H4A	120.8	C13—C12—C11	121.31 (9)
C3—C4—H4A	120.8	C13—C12—H12A	119.3
F2—C5—C4	118.12 (8)	C11—C12—H12A	119.3
F2—C5—C6	118.35 (8)	C12—C13—C8	119.51 (9)
C4—C5—C6	123.53 (9)	C12—C13—H13A	120.2
C1—C6—C5	115.22 (8)	C8—C13—H13A	120.2
C1—C6—C7	123.11 (8)	C11—C14—C11^i^	115.22 (11)
C5—C6—C7	121.53 (8)	C11—C14—H14A	108.5
O1—C7—N1	125.27 (9)	C11^i^—C14—H14A	108.4
O1—C7—C6	119.92 (8)		
			
F1—C1—C2—C3	179.76 (9)	C5—C6—C7—O1	−46.84 (13)
C6—C1—C2—C3	−1.62 (15)	C1—C6—C7—N1	−50.49 (12)
C1—C2—C3—C4	0.95 (16)	C5—C6—C7—N1	133.95 (9)
C2—C3—C4—C5	0.57 (16)	C7—N1—C8—C9	42.34 (14)
C3—C4—C5—F2	178.73 (9)	C7—N1—C8—C13	−140.39 (10)
C3—C4—C5—C6	−1.60 (15)	C13—C8—C9—C10	−0.27 (15)
F1—C1—C6—C5	179.29 (8)	N1—C8—C9—C10	176.96 (9)
C2—C1—C6—C5	0.68 (14)	C8—C9—C10—C11	−0.61 (15)
F1—C1—C6—C7	3.47 (13)	C9—C10—C11—C12	1.22 (15)
C2—C1—C6—C7	−175.14 (9)	C9—C10—C11—C14	−176.46 (9)
F2—C5—C6—C1	−179.35 (8)	C10—C11—C12—C13	−0.98 (15)
C4—C5—C6—C1	0.98 (14)	C14—C11—C12—C13	176.68 (9)
F2—C5—C6—C7	−3.46 (13)	C11—C12—C13—C8	0.14 (15)
C4—C5—C6—C7	176.87 (9)	C9—C8—C13—C12	0.50 (15)
C8—N1—C7—O1	0.91 (16)	N1—C8—C13—C12	−176.80 (9)
C8—N1—C7—C6	−179.94 (8)	C12—C11—C14—C11^i^	47.96 (8)
C1—C6—C7—O1	128.72 (10)	C10—C11—C14—C11^i^	−134.43 (10)

Symmetry codes: (i) −*x*, *y*, −*z*+1/2.

### Hydrogen-bond geometry (Å, °)

**Table:**

*D*—H···*A*	*D*—H	H···*A*	*D*···*A*	*D*—H···*A*
N1—H1N1···O1^ii^	0.841 (15)	2.078 (15)	2.8811 (11)	159.4 (14)
C9—H9A···F1^iii^	0.95	2.41	3.2318 (11)	145

Symmetry codes: (ii) *x*, −*y*+2, *z*−1/2; (iii) *x*, −*y*+2, *z*+1/2.
